# Expression and regulation of CCL18 in synovial fluid neutrophils of patients with rheumatoid arthritis

**DOI:** 10.1186/ar2294

**Published:** 2007-09-17

**Authors:** Judith Auer, Markus Bläss, Hendrik Schulze-Koops, Stefan Russwurm, Thomas Nagel, Joachim R Kalden, Martin Röllinghoff, Horst Ulrich Beuscher

**Affiliations:** 1 Institute for Clinical Microbiology, Immunology and Hygiene, University of Erlangen-Nuremberg, Wasserturmstrasse 3-5, D-91054 Erlangen, Germany; 2 SIRS-Lab GmbH, Winzerlaer Strasse 2, D-07745 Jena, Germany; 3 Department of Internal Medicine III and Institute for Clinical Immunology, Rheumatology and Onkology, University of Erlangen-Nuremberg, Krankenhausstrasse 12, D-91054 Erlangen, Germany; 4 Nikolaus Fiebiger Centre for Molecular Medicine, Clinical Research Group III, University of Erlangen-Nuremberg, Glücksstrasse 5, D-91054 Erlangen, Germany; 5 Clinics of Anesthesiology and Intensive Therapy, Friedrich-Schiller-University of Jena, Bachstrasse 18, D-07743 Jena, Germany

## Abstract

Rheumatoid arthritis (RA) is characterized by the recruitment of leukocytes and the accumulation of inflammatory mediators within the synovial compartment. Release of the chemokine CCL18 has been widely attributed to antigen-presenting cells, including macrophages and dendritic cells. This study investigates the production of CCL18 in polymorphonuclear neutrophils (PMN), the predominant cell type recruited into synovial fluid (SF). Microarray analysis, semiquantitative and quantitative reverse transcriptase polymerase chain reaction identified SF PMN from patients with RA as a novel source for CCL18 in diseased joints. Highly upregulated expression of other chemokine genes was observed for CCL3, CXCL8 and CXCL10, whereas CCL21 was downregulated. The chemokine receptor genes were differentially expressed, with upregulation of CXCR4, CCRL2 and CCR5 and downregulation of CXCR1 and CXCR2. In cell culture experiments, expression of CCL18 mRNA in blood PMN was induced by tumor necrosis factor α, whereas synthesis of CCL18 protein required additional stimulation with a combination of IL-10 and vitamin D_3_. In comparison, recruited SF PMN from patients with RA were sensitized for CCL18 production, because IL-10 alone was sufficient to induce CCL18 release. These results suggest a release of the T cell-attracting CCL18 by PMN when recruited to diseased joints. However, its production is tightly regulated at the levels of mRNA expression and protein synthesis.

## Introduction

Polymorphonuclear neutrophils (PMN) are effector cells during inflammation, and their migration to sites of infection is essential in controlling microbial growth and dissemination [1,2]. Neutrophilic infiltration has, however, also been implicated in the pathology of various acute and chronic inflammatory diseases, such as rheumatoid arthritis (RA), gouty arthritis and Crohn's disease [3-5]. In RA, PMN are highly abundant in synovial fluid (SF) during acute flares of the disease [6]. In addition, PMN have been detected at the pannus–cartilage junction at sites of erosion, suggesting that they contribute to cartilage destruction through the release of their proteolytic contents [7,8]. Moreover, PMN obtained from SF from patients with RA were found to produce a number of cytokines and chemotactic factors involved in the recruitment of inflammatory cells [9,10]. Because PMN are among the first cells to arrive at an inflammatory site, these observations raise the possibility that SF PMN may be able to perpetuate the inflammatory process through the release of inflammatory mediators such as cytokines and chemokines.

Chemokines are a superfamily of more than 50 different chemotactic proteins participating in the cellular traffic of immune and inflammatory responses [11]. They are categorized into at least four subfamilies, namely C, CC, CXC and CX3C, distinguished by the presence or absence of a residue (X) between two conserved cysteine residues in the N terminus. Chemokines range in size from 8 to 10 kDa and are produced by a wide variety of cell types [10,12]. Their production either occurs constitutively or may be induced by appropriate stimulation with exogenous or endogenous agents, such as the proinflammatory cytokines IL-1 and TNF-α [13]. Chemokines are known to exert their biological effects on various cell types through binding to G-protein-coupled cell surface receptors with seven transmembrane domains [14]. Chemokine receptors may be specific for one ligand or they may bind several chemokines [15], thus allowing redundancy of the system. During activation of PMN, expression of CC chemokine receptors is upregulated, whereas that of some CXC receptors is downregulated [16]. Regulation of chemokine activities therefore occurs at the levels of receptor expression as well as ligand production.

CCL18, also named pulmonary and activation-regulated chemokine (PARC), dendritic cell-derived CC chemokine-1 (DC-CK1), alternative macrophage activation-associated CC chemokine-1 (AMAC-1) and macrophage inflammatory protein-4 (MIP-4), has been described to attract naïve T cells and mantle-zone B cells [17]. Cellular sources of CCL18 are primarily monocytes/macrophages and dendritic cells (DCs). Its production occurs constitutively but may be increased by additional stimulation with cytokines such as IL-10 and IL-4 as well as vitamin D_3 _[18,19]. In RA, expression of mRNA encoding CCL18 was observed in synovial tissue and it coincided with CCL18 accumulation in SF [19,20].

The present study used a broad-scale experimental approach, involving microarray and quantitative RT-PCR analyses, to determine whether PMN can serve as a site for CCL18 production in RA. The results demonstrate that SF PMN from patients with RA are a cellular source for CCL18, the production of which is differentially regulated at the levels of mRNA expression and protein synthesis. Moreover, because PMN are recruited into SF, a characteristic chemokine expression profile is induced with highly upregulated mRNAs for CCL3, CCL18, CXCL8 and CXCL10 and downregulation of CCL21 mRNA.

## Materials and methods

### Patients

SF samples were taken from knees of nine patients with active RA, for treatment and diagnostic purposes. Aliquots of these samples and EDTA-treated blood from these patients were used in this study after informed consent had been obtained. Four patients were receiving anti-TNF-α therapy, three were being treated with conventional therapy (nonsteroidal anti-inflammatory drugs, steroids or disease-modifying anti-rheumatic drugs), and two patients were receiving no medication at the time of synovial effusion although those had been treated earlier (Table 1). Hence, all patients did not respond sufficiently to the therapeutic treatment.

**Table 1:** Clinical data for patients with RA and healthy donors providing samples for microarray analysis

Subject	Sex	Age (years)	Medication
RA patient			
1	Female	55	NSAID (diclofenac)
2	Female	53	None
3	Female	72	Anti-TNF-α (adalimumab)
4	Female	ND	None
5	Female	44	Anti-TNF-α (infliximab)
6	Female	80	DMARDs (methotrexate and resochine)
7	Female	35	Anti-TNF-α (infliximab)
8	Male	61	Anti-TNF-α (infliximab)
9	Male	51	DMARD (methotrexate); steroid (cortisone)
Healthy donor			
1	Female	30	None
2	Female	26	None
3	Female	38	None
4	Male	27	None

RA, rheumatoid arthritis; NSAID, nonsteroidal anti-inflammatory drug; DMARD, disease-modifying anti-rheumatic drug; ND, not determined.

### Purification of mononuclear cells and PMN from blood and SF

Purification of mononuclear cells (peripheral blood mononuclear cells; PBMC) and PMN from blood was performed as described [21], with minor modifications. For sedimentation of erythrocytes, 1 volume of EDTA-treated blood was incubated with 1 volume of 3% dextran T500 (Roth, Karlsruhe, Germany) in PBS (Biochrom, Berlin, Germany) for 20 minutes at 4°C. The leucocyte-rich supernatant was centrifuged at 500 *g *for 10 minutes at 6°C in a Multifuge (Heraeus, Hanau, Germany). The pellet was resuspended in PBS, overlaid on an isotonic discontinous Percoll gradient (Amersham Biosciences, Freiburg, Germany) with densities of 1.075 g/ml and 1.09 g/ml and centrifuged at 750 *g *for 25 minutes at 6°C. PBMC or PMN were collected at the relevant interphase and washed twice with PBS. With the exception of dextran sedimentation, the preparation of SF PMN was performed similarly. Cell-free SF was obtained by centrifugation of SF at 750 *g *for 25 minutes and stored at -70°C in aliquots until use. PMN and mononuclear cells were counted in SF from patients with RA. The ratio between PMN and mononuclear cells was approximately 1:10.

### Flow cytometry

The purity of PMN was routinely analyzed by flow cytometry with FACSCalibur (BD Biosciences, Heidelberg, Germany). In brief, to prevent nonspecific binding, 3 × 10^5 ^cells were preincubated for 10 minutes with heat-inactivated (20 minutes, 56°C) human serum and were then incubated with a combination of fluorescein isothiocyanate-conjugated CD66b antibody (Immunotech, Hamburg, Germany) and allophycocyanin (APC)-conjugated CD14 antibody (Caltag, Hamburg, Germany). After 30 minutes at 4°C, cells were washed twice with PBS/1% FCS (Sigma, Deisenhofen, Germany) and kept on ice until analysis. Data were analyzed with CellQuest software (BD Biosciences), revealing a purity of 98 to 99% CD66b-positive PMN with a contamination of less than 0.05% CD14-positive and CD66b-negative cells. Analysis of PMN preparations of three donors with anti-CD56-APC (BD Biosciences), anti-CD3-phycoerythrin (Caltag) and anti-CD19-APC (Caltag) revealed 0.01 to 0.04% natural killer cells, 0.06 to 0.33% T cells and up to 0.06% B cells. In addition, PMN preparations were subjected to histochemistry (Diff-Quick staining; Dade Behring, Düdingen, Switzerland) showing 1 to 3% eosinophils in each preparation of blood PMN. Selected cell culture experiments were performed with PMN preparations depleted of eosinophils with anti-CD16 antibodies by means of magnetic-activated cell sorting (Miltenyi Biotec, Bergisch Gladbach, Germany) to exclude the possibility that contaminating eosinophils accounted for CCL18 production. Notably, no eosinophils were detectable in preparations of SF PMN.

### Cell culture

For culturing PMN, RPMI 1640 containing L-glutamine and sodium bicarbonate (Sigma) was supplemented with 5 mM HEPES (Sigma), 100 IU/ml penicillin, 100 μg/ml streptomycin (Sigma) and 10% FCS. For pretreatment of PMN with SF, blood PMN from healthy donors were seeded in six-well-plates (Corning Costar, Bodenheim, Germany) at a density of 5 × 10^7 ^cells in 5 ml of culture medium or 2.5 ml of culture medium plus 2.5 ml of SF from patients with RA. After 10 hours of incubation, cells were washed twice. Pretreated blood PMN as well as freshly isolated blood PMN from healthy donors or blood and SF PMN from patients with RA were seeded in 24-well plates (Greiner Bio-One GmbH, Frickenhausen, Germany) at a density of 5 × 10^6 ^cells in 500 μl of culture medium with and without 20 ng/ml IL-10 (Endogen, Eching, Germany), 10^-7 ^M vitamin D_3 _(1,25-dihydroxycholecalciferol; Calbiochem, Darmstadt, Germany) and different concentrations (10, 1, 0.5 and 0.25 ng/ml) of recombinant TNF-α (Biolegend, Eching, Germany). In addition, for co-culture experiments, 5 × 10^5 ^EA-hy.926 human endothelial cells (Department of Experimental Pathology, St Bartholomew's and the Royal London School of Medicine, London) were plated in 24-well plates in DMEM with 4,500 mg/l glucose, L-glutamine, sodium bicarbonate and pyridoxine hydrochloride (Sigma) supplemented with 100 IU/ml penicillin, 100 μg/ml streptomycin and 10% FCS. After 1 hour of incubation at 37°C, 5% CO_2 _and 95% humidity, 5 × 10^5 ^PBMC or 5 × 10^6 ^blood PMN from healthy donors were added to a final volume of 500 μl of DMEM/10% FCS. Additionally PMN and EA-hy.926 cells were cultured in Boyden chambers, separated by a transwell membrane with a pore size of 0.4 μm (Corning Costar). For control experiments, blood PMN or EA-hy.926 cells were γ-irradiated (40 Gy), washed twice after 24 hours of incubation and co-cultured with vital EA-hy.926 cells or PMN, respectively. After 24 or 48 hours of incubation, cells and supernatants were prepared for subsequent isolation of RNA or ELISA analysis.

### RNA sample preparation

For microarray analysis and RT-PCR, total RNA was extracted from PMN with RNeasy Mini Spin Columns (Qiagen, Hilden, Germany) in accordance with the manufacturer's instructions. From cell cultures, RNA was prepared with the acid-phenol extraction procedure [22]. All RNA samples were digested with DNAse (DNA-free™ Kit; Ambion, Huntingdon, UK). RNA yields were determined spectrophotometrically by measuring the absorbance at 260 and 280 nm. All RNA samples used for microarray analysis were analyzed with an RNA 6000 Nano Labchip (Agilent Technologies, Böblingen, Germany) for RNA degradation. Only RNA preparations with no detectable degradation were used for microarray analysis.

### Microarray hybridization

Experiments were performed with the Lab-Arraytor^®^-60 inflammation microarray (SIRS-Lab, Jena, Germany) comprising 800 probes (each measurement being made in triplicate) addressing 780 transcripts corresponding to inflammation as well as 20 control probes. The microarray data according to Minimal Information about a Microarray Experiment (MIAME) guidelines were deposited in the database ArrayExpress [23] with the accession number E-MEXP-994. RNA samples of SF PMN from nine RA patients and of blood PMN from four healthy donors (Table 1) were amplified with BD Atlas™ SMART™ Fluorescent Amplification Kit (BD Biosciences) in accordance with the manufacturer's instructions. cDNAs were cleaned with a Promega Wizard PCR clean-up Kit (Promega, Mannheim, Germany) and labeled by use of the Dyomics DY-648-S-NHS/DY-548-S-NHS dye system (Dyomics, Jena, Germany). Dyomics DY-648-S-NHS-labeled cDNA was cohybridized with DY-548-S-NHS-labeled cDNA obtained from the same amount of total RNA isolated from the immature monocytic cell line SigM5 obtained from the German Resource Centre for Biological Material (DSMZ, Braunschweig, Germany) and subjected to culture under standard conditions. After incubation in a hybridization apparatus (HS 400; TECAN Group Ltd, Männedorf, Switzerland), for 10 hours at 42°C in a formamide-based hybridization buffer system, arrays were washed in accordance with the manufacturer's instructions and dried; hybridization signal intensities were measured immediately with an array scanner (model Gene Pix 4000B; Axon Instruments, Union City, CA, USA).

### Microarray data preprocessing

Digital images resulting from post-hybridization array scanning were quantified with GenePix Pro 4.0 software (Axon Instruments). GenePix™ Analysis Software was used for spot detection and quantification and also for spot quality flagging. Significantly regulated genes were determined by using a two-sample permutation test. The threshold of significance for multiple comparisons was defined with calculating the corresponding *q *value for each *p *value [24]. For normalization and variance-stabilized transformation of raw signals the method of Huber and colleagues [25] was used, in which the data were transformed by an arcsinh function; for example, a transformed ratio of ± 0.4 corresponds to approximately 1.5-fold change (almost identical with the natural logarithm). Genes with mean transformed ratios less than -1 and larger than +1 were significantly regulated. Genes were clustered with the Database for Annotation, Visualization and Integrated Discovery (DAVID) software [26].

### Semiquantitative and quantitative RT-PCR

Changes in gene expression assessed by microarray analysis were confirmed by semiquantitative and quantitative RT-PCR for selected genes, namely CXCL10, CCL18, CCRL2 and CXCR4. In brief, cDNA was synthesized from 0.5 μg of total RNA by using 0.5 μg of oligo(dT) 16-mer primer (Thermo, Ulm, Germany), 0.5 mM dNTP (Invitrogen, Karlsruhe, Germany) and 1 unit (U) of Omniscript reverse transcriptase (Qiagen) in a final volume of 25 μl at 37°C for 60 minutes and at 93°C for 5 minutes. PCR was performed in a 25 μl reaction mixture containing 2 μl of cDNA sample, 0.625 U of *Taq *polymerase (Qiagen), 0.2 mM dNTPs (Invitrogen) and sense and antisense primers (each at 0.4 μM). Amplification was performed at 95°C for 2 minutes followed by 34 cycles with a denaturing step at 94°C for 1 minute, an annealing step at 55°C for 50 s and an extension step at 72°C for 90 s. A final extension step was performed at 72°C for 7 minutes on a thermocycler (Biozym, Hess. Oldendorf, Germany). Primers for CCRL2 (5'-TGA CAA GTA TGA CGC CCA G-3' and 5'-ACC AGG ATA AGC ACA ACC AG-3'), CCL18 (5'-CTC CTT GTC CTC GTC TGC AC-3' and 5'-TCA GGC ATT CAG CTT CAG GT-3'), CXCR4 [27], CXCL10 [28] and β-actin [29] were purchased from MWG Biotech (Ebersberg, Germany). PCR samples were separated on 2% agarose gels (Roth), revealed by ethidium bromide staining (Merck, Darmstadt, Germany) and photographed on an ImageMaster VDS (Amersham Biosciences).

Quantitative PCR was performed in a 20 μl reaction mixture containing 2 μl of cDNA (diluted 1:5 in water), 1 U of Platinum-Taq-Polymerase (Invitrogen), 0.5× SYBR-Green (Roche, Mannheim, Germany), 5% dimethylsulfoxide (Sigma), 0.5 mg/ml bovine serum albumin (New England Biolabs, Frankfurt, Germany), 0.25 mM dNTP (Amersham), 4 mM magnesium chloride (Invitrogen) and sense and antisense primers (each at 0.4 μM). Amplification was performed with a real-time light cycler (Roche) at 95°C, 10 minutes, 20°C/s of preincubation followed by 50 cycles of denaturing at 95°C, 15 s, 20°C/s, annealing at 55°C (CCL18) and 56°C (HPRT), 10 s, 20°C/s and extension at 72°C, 15 s, 20°C/s. The melting curve was performed at 95°C, 0 s, 20°C/s followed by 65°C, 15 s, 20°C/s and 95°C, 0 s, 0.1°C/s. Cooling was done at 40°C, 30 s, 20°C/s. CCL18 values were normalized with HPRT values and are presented as relative gene expression ratios with the 2^-ΔΔCt ^method [30]. CCL18 primer (5'-GGG GGC TGG TTT CAG AAT A-3' and 5'-CTC CTT GTC CTC GTC TGC AC-3') and HPRT primer (5'-GAC TTT GCT TTC CTT GGT CA-3' and 5'-GGC TTT GTA TTT TGC TTT TCC-3') for quantitative RT-PCR were purchased from MWG Biotech. All primer sequences correspond to sequences for human cDNAs deposited in GenBank.

### ELISA analysis

CCL18 and TNF-α levels in culture supernatants were quantified with human CCL18 and TNF-α ELISA (DuoSet^® ^ELISA Development System; R&D Systems, Wiesbaden-Nordenstadt, Germany) in accordance with the manufacturer's instructions. Substrate reagents A and B were from BD Biosciences. The sensitivity of both assays was 5 pg/ml.

### Statistical analysis

All experiments were performed at least three times. The values are expressed as means ± SEM. A Wilcoxon test was used to assess the significance of differences between two conditions. All *p *values are two-tailed, and *p *< 0.05 is considered significant. Statistical analysis was performed with the software package provided by Prism 3.0^®^.

## Results

### Expression of CCL18 in SF PMN

**Figure 1:**
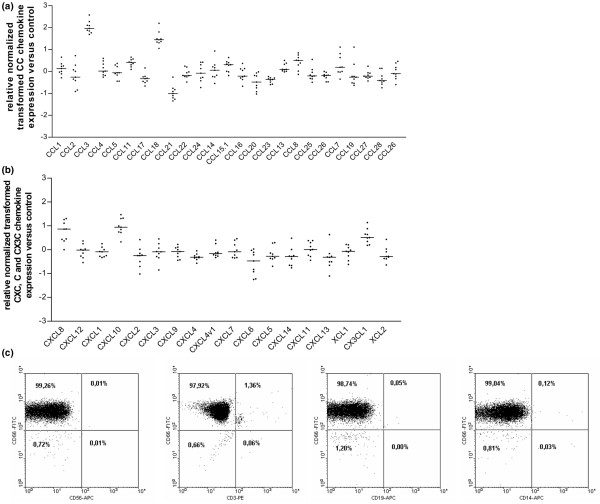
Microarray analysis of chemokine gene expression in synovial fluid polymorphonuclear neutrophils. RNA of SF synovial fluid polymorphonuclear neutrophils (SF PMN) from nine patients with rheumatoid arthritis (RA) was analyzed for the expression of CC chemokines **(a) **and CXC, C and CX3C chemokines **(b)**. Data obtained by Gene Pix™ Analysis Software were normalized, transformed and denoted as *x*-fold regulation versus the expression of blood PMN from healthy donors. Bars represent the median expression between nine RNA samples. Genes with median expression ratios less than -1 or more than +1 were significantly regulated. **(c) **SF PMN from one representative patient with RA (no. 2) were subjected to flow cytometry with fluorescein isothiocyanate-conjugated anti-CD66b, allophycocyanin (APC)-conjugated anti-CD56, phycoerythrin (PE)-conjugated anti-CD3, anti-CD19-APC and anti-CD14-APC antibodies.

**Figure 2:**
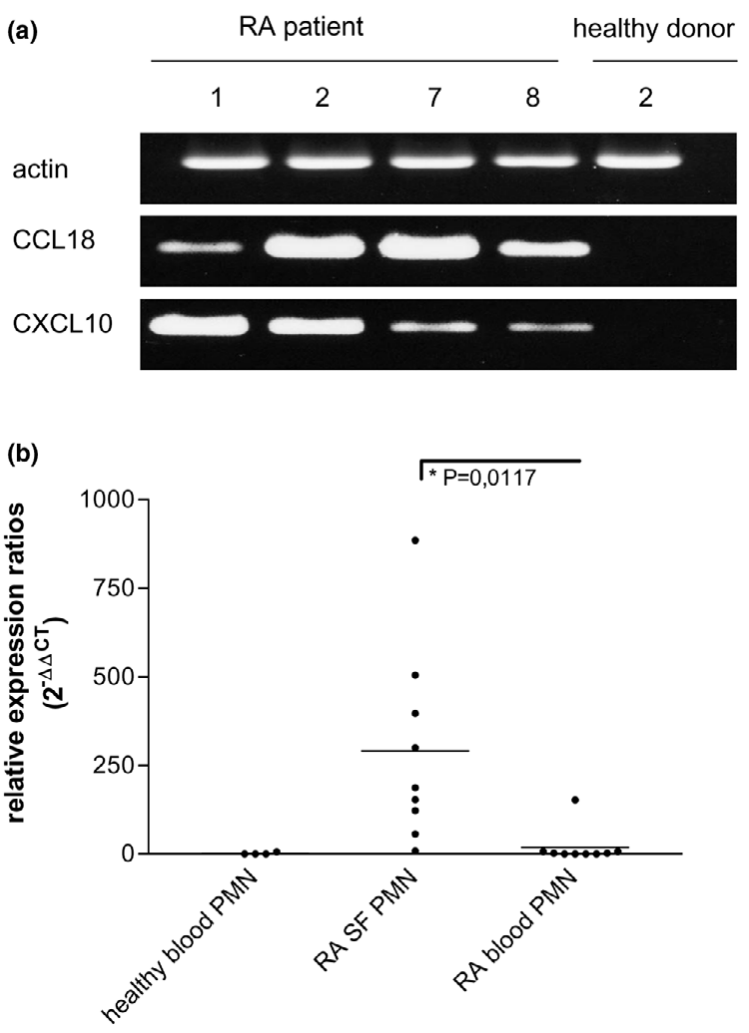
Semiquantitative and quantitative RT-PCR analysis of CCL18 mRNA in polymorphonuclear neutrophils. **(a) **Total RNA from synovial fluid polymorphonuclear neutrophils (SF PMN) of rheumatoid arthritis (RA) patients nos 1, 2, 7 and 8 and from blood PMN from healthy donor no. 2 was amplified by semiquantitative RT-PCR with primers for CCL18, CXCL10 and actin and subjected to agarose gel electrophoresis. PCR was repeated twice. **(b) **Total RNA from blood and SF PMN from patients with RA (*n *= 9) and from blood PMN from healthy donors (*n *= 4) were subjected to quantitative RT-PCR with primers for CCL18 and HPRT. CCL18 transcript levels are presented as relative expression ratios. Bars represent median expression between RNA samples.

RNA preparations were obtained from SF PMN from nine individual patients with RA and from peripheral blood PMN from four healthy donors (Table 1) and subjected to microarray analysis. Results summarized in Figure 1 show a highly upregulated expression of CCL3 and CCL18 mRNAs, a downregulation of CCL21 mRNA (Figure 1a) and an upregulation of CXCL8 and CXCL10 mRNAs (Figure 1b) in SF PMN from each patient in comparison with blood PMN from healthy donors. As revealed by flow cytometry with CD66b antibodies, SF PMN form one representative RA patient prepared for microarray experiments were of 99% purity. Natural killer cells, T cells, B cells and monocytes/macrophages were not detectable (Figure 1c). To validate the microarray data, semiquantitative RT-PCR was performed with the same RNAs as used in the microarray analysis of four randomly selected RA patients (namely nos 1, 2, 7 and 8) and one of the healthy donors (namely no. 2). As shown in Figure 2a, mRNA expression for two selected chemokines, CCL18 and CXCL10, was readily detectable in SF PMN but remained undetectable in peripheral blood PMN from the healthy donor. In addition, quantitative RT-PCR revealed changes in CCL18 expression in SF PMN from RA patients (*n *= 9; Figure 2b) similar to those observed with microarray analysis. These data indicate that the microarray analysis reflects a true transcriptional profile of chemokines in SF PMN. Differences observed in CCL18 mRNA expression levels in SF PMN between individual RA patients did not relate to the actual therapeutic treatment (*p *= 0.2612, none versus conventional therapy; *p *= 0.3798, none versus anti-TNF therapy) as determined by quantitative RT-PCR (data not shown). Notably, no significant levels of CCL18 mRNA were detectable in blood PMN form RA patients (*n *= 9; Figure 2b), suggesting that CCL18 gene expression in SF PMN occurs as a result of the recruitment of PMN into the inflammatory milieu of the joint.

### Differential expression of chemokine receptors in SF PMN

**Figure 3:**
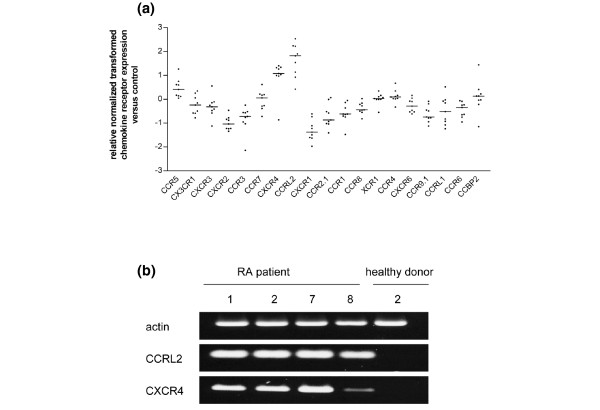
Microarray analysis of chemokine receptor gene expression in synovial fluid polymorphonuclear neutrophils. **(a) **RNA of synovial fluid polymorphonuclear neutrophils (SF PMN) from nine patients with rheumatoid arthritis (RA) was analyzed for chemokine receptor expression. Data obtained by Gene Pix™ Analysis Software were normalized, transformed and denoted as *x*-fold regulation versus expression of blood PMN from healthy donors. Bars represent median expression between nine RNA samples. Genes with median expression ratios less than -1 or more than +1 were significantly regulated. **(b) **Total RNA from SF PMN from RA patients nos 1, 2, 7 and 8 and from blood PMN from healthy donor no. 2 was amplified by semiquantitative RT-PCR with primers for CCRL2, CXCR4 and actin and subjected to agarose gel electrophoresis. PCR was repeated twice.

To estimate changes in the responsiveness of SF PMN to chemokine ligands, expression levels of chemokine receptor mRNAs were determined by microarray analysis as described above. Results in Figure 3a show an upregulation of CXCR4, CCRL2 and CCR5 in SF PMN from patients with RA in comparison with blood PMN from healthy donors. For many other receptors, particularly the neutrophil receptors CXCR1 and CXCR2, mRNA levels were downregulated. When RNA from SF PMN obtained from RA patient nos 1, 2, 7 and 8 and healthy donor no. 2 was used, semiquantitative RT-PCR (Figure 3b) revealed an upregulation of two selected chemokine receptors, CCRL2 and CXCR4, in SF PMN, thereby confirming the results of the microarray analysis. These data therefore indicate that chemokine responsiveness of SF PMN is regulated, at least in part, through the differential regulation of chemokine receptor expression.

### Induction of CCL18 protein synthesis in SF PMN

**Figure 4:**
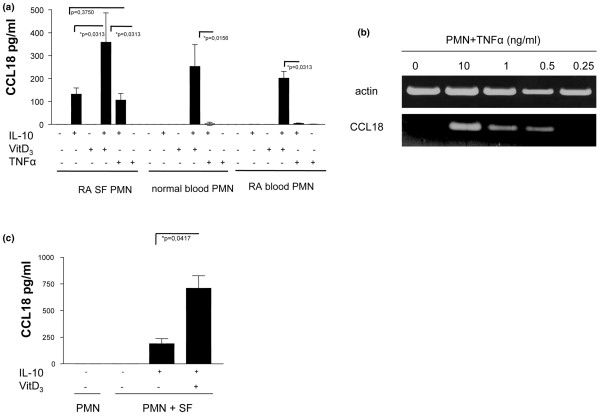
Induction of CCL18 mRNA and protein in synovial fluid and blood polymorphonuclear neutrophils. **(a) **Synovial fluid polymorphonuclear neutrophils (SF PMN) of patients with rheumatoid arthritis (RA) and blood PMN from healthy donors and patients with RA were incubated with 20 ng/ml IL-10, 10^-7 ^M vitamin D_3 _and 10 ng/ml TNF-α for 48 hours. Levels of CCL18 in the supernatant were determined by ELISA. Data represent the geometric mean ± SEM for three independent experiments performed in duplicate. **(b) **PMN from healthy donors were incubated for 24 hours with various amounts of recombinant TNF-α. Total RNA was amplified by semiquantitative RT-PCR with primers for CCL18 and actin and subjected to agarose gel electrophoresis. This result is representative of three independent experiments with PMN from three different donors. **(c) **Blood PMN from healthy donors were incubated in the presence or absence of SF from patients with RA for 10 hours, then washed twice and incubated for a further 48 hours with 20 ng/ml IL-10 and 10^-7 ^M vitamin D_3_. Data represent the geometric mean ± SEM for three independent experiments performed in duplicate.

To determine whether SF PMN might contribute to CCL18 protein levels in inflamed joints, supernatants of cultured SF PMN from patients with RA were subjected to ELISA analysis. The results (Figure 4a) showed no spontaneous release of CCL18 by SF PMN. However, CCL18 could be induced by stimulation of SF PMN with IL-10; indeed, IL-10 in combination with vitamin D_3 _even enhanced CCL18 release. In contrast, SF PMN from patients with RA did not release CCL18 in response to TNF-α. In addition, the effect of IL-10 in combination with TNF-α was not significantly different from that of IL-10 alone, suggesting that TNF-α may not be involved in CCL18 protein synthesis. Because IL-10 is frequently found in SF from patients with RA [31], PMN recruited into joints are likely to contribute to the local production of CCL18. Furthermore, because SF PMN from patients with RA were shown to express CCL18 mRNA in the absence of detectable CCL18 protein, it is evident from these data that the synthesis of CCL18 protein requires a secondary signal.

### TNF-α induces CCL18 mRNA in the absence of CCL18 protein in blood PMN

To identify regulatory mechanisms involved in CCL18 expression, a putative role of TNF-α as a sensitizing agent was investigated in cultures of blood PMN from healthy donors. The results shown in Figure 4b confirmed TNF-α as a potent inducer of CCL18 mRNA expression. However, ELISA analysis of CCL18 levels in supernatants of the same cell cultures revealed no detectable CCL18 protein after 24 or 48 hours of incubation (data not shown), indicating that TNF-α induced CCL18 mRNA expression in the absence of protein synthesis. Interestingly, as shown in Figure 4a, neither blood PMN from healthy donors nor blood PMN from patients with RA released significant amounts of CCL18 in response to IL-10 alone or to a combination of IL-10 and TNF-α, suggesting that TNF-α alone may not be sufficient to prime PMN for CCL18 synthesis. This conclusion is supported by data showing that CCL18 production could be induced by stimulating PMN with IL-10 and vitamin D_3 _in the absence of exogenous TNF-α (Figure 4a). To investigate whether the difference between SF PMN and blood PMN in their responsiveness to IL-10 was due to soluble factors present in SF, blood PMN from healthy donors were pretreated with SF from patients with RA and subsequently stimulated with IL-10 alone or in combination with vitamin D_3_. The results in Figure 4c show that preincubation with SF supported induction by IL-10 alone. Incubation with both IL-10 and vitamin D_3 _produced the release of even more CCL18 by PMN preincubated with SF.

### Endothelial cells induce CCL18 protein in PMN

**Figure 5:**
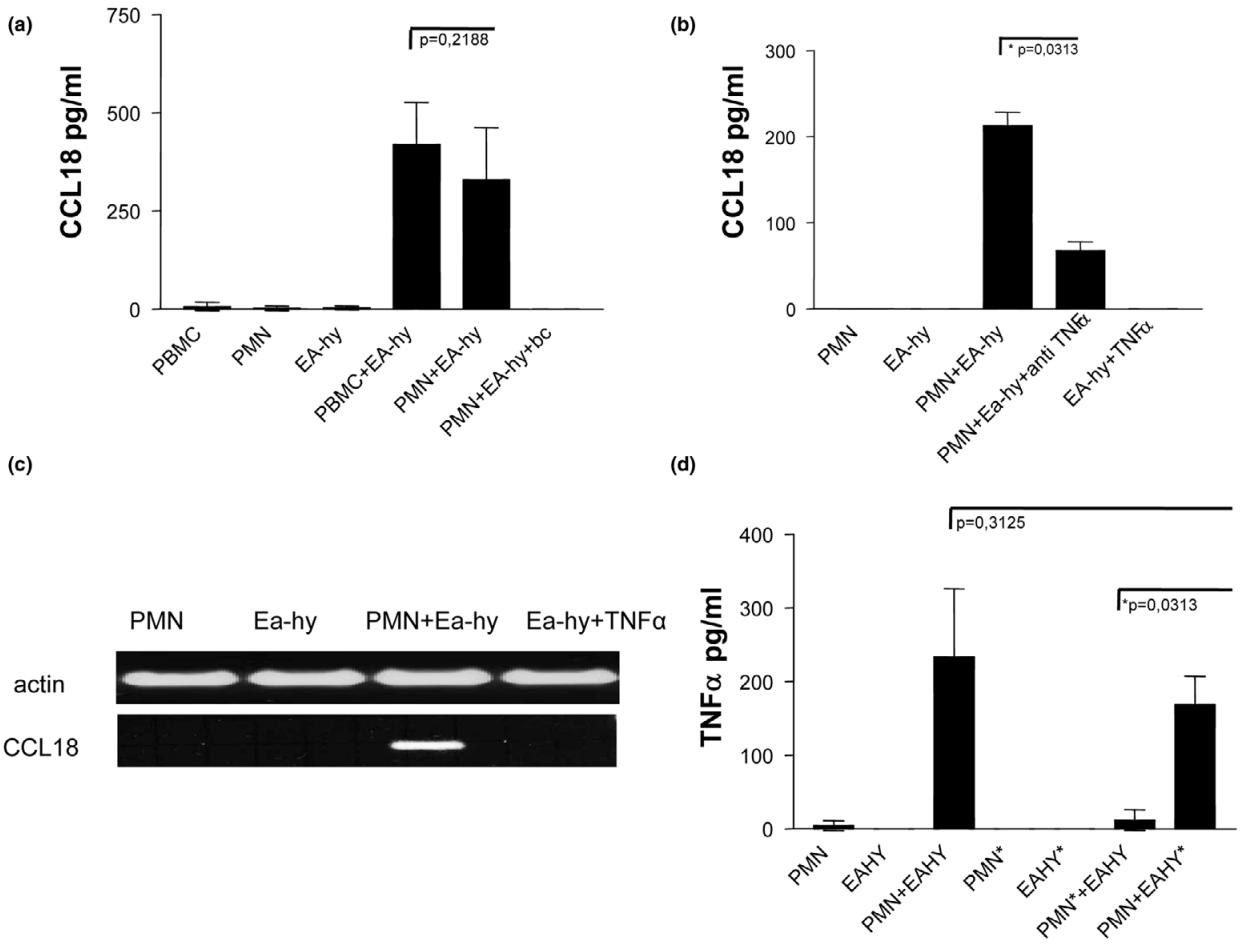
CCL18 induction of polymorphonuclear neutrophils co-cultured with endothelial cells requires cell–cell contact and TNF-α. **(a) **CCL18 levels were measured by ELISA in culture supernatants of 5 × 10^5 ^peripheral blood mononuclear cells, 5 × 10^6 ^polymorphonuclear neutrophils (PMN) or 5 × 10^5 ^EA-hy.926 cells alone or after co-culture with EA-hy.926 cells after incubation for 48 hours. Direct cell–cell contact of PMN and EA-hy.926 cells was prevented by Boyden chambers (bc) as indicated. Data represent the geometric mean ± SEM of CCL18 measured in four independent experiments performed in duplicate. **(b) **CCL18 levels were measured in culture supernatants of PMN and EA-hy.926 cells alone or after co-culture of both cell types after incubation for 48 hours. Cultures were supplemented with anti-TNF-α antibodies (infliximab; 50 μg/ml) or 10 ng/ml TNF-α as indicated. Data represent the geometric mean ± SEM of three independent experiments performed in duplicate. **(c) **RNA was prepared from PMN and EA-hy.926 cells alone or after co-culture of both cell types after incubation for 24 hours. Cultures were supplemented with TNF-α (10 ng/ml) as indicated. RNA samples were amplified by semiquantitative RT-PCR with primers for CCL18 and actin and subjected to gel electrophoresis. This result is representative of three independent experiments with PMN from three different donors. **(d) **TNF-α levels were measured by ELISA in culture supernatants of PMN and EA-hy.926 cells alone and in co-cultures of PMN and EA-hy.926 cells before or after γ-irradiation (40 Gy) of one of these cell types. The irradiated cell type is marked with an asterisk. Data represent the geometric mean ± SEM for three independent experiments performed in duplicate.

To determine whether extravasation might have a role in the induction of CCL18 release, PMN and PBMC from healthy donors were co-cultured with the endothelial cell line EA-hy.926. Results in Figure 5a revealed high levels of CCL18 in culture supernatants of PMN or PBMC co-cultured with endothelial cells, whereas no release of CCL18 was observed when PMN and EA-hy.926 cells were incubated separately from each other in Boyden chambers. Similar levels of CCL18 were released into supernatant of RA blood or SF PMN co-cultured with endothelial cells (data not shown). The data therefore indicate that cell–cell contact is crucial for the induction of CCL18. Release of CCL18 was induced by endogenous TNF-α, as revealed by blocking experiments with excess anti-TNF-α antibodies (Figure 5b). In controls, stimulation of EA-hy.926 cells with TNF-α was found to induce neither the release of CCL18 (Figure 5b) nor the expression of CCL18 mRNA (Figure 5c), suggesting that CCL18 derives from PMN. To determine the origin of TNF-α in co-cultures, either PMN or EA-hy.926 cells were pretreated with γ-radiation and then incubated with the viable cellular counterpart. As shown in Figure 5d, irradiation of PMN on their own abolished the production of TNF-α in co-culture with EA-hy.926 cells, indicating that PMN are the predominant source of TNF-α in co-cultures. The results therefore suggest that cell contact with endothelial cells induces TNF-α in PMN, which in turn induces CCL18 production by PMN in an autocrine manner.

## Discussion

This study describes PMN, and particularly SF PMN from patients with RA, as a novel cellular source of the chemokine CCL18. As judged by the immunostaining of SF PMN with anti-CD66b, nongranulocytic cells constituted less than 1% of the PMN preparations. Flow cytometry confirmed the low frequency (0.05%) of CD14-positive monoctyes/macrophages in isolated PMN populations, suggesting that contaminating cells are unlikely to have a significant role in the chemokine profile determined by microarray analysis. In addition, data are presented to show that the production of CCL18 by PMN is differentially regulated at the levels of mRNA expression and protein synthesis. CCL18 was originally described to be constitutively expressed in lung and lymphoid tissue [32]. At the cellular level CCL18 production seems to be restricted to dendritric cells and monocytes/macrophages [18,19]. T helper cell type 2-related cytokines, including IL-4, IL-10 and IL-13, were found to induce or enhance CCL18 expression, whereas the T helper type 1-derived interferon-γ suppressed CCL18 mRNA expression [17]. There is also evidence for a superantigen-induced CCL18 production by PBMC [19].

It is shown here that CCL18 mRNA expression is induced in PMN by TNF-α in the absence of detectable amounts of CCL18 protein, suggesting that TNF-α provides an early signal for CCL18 transcription. Consistent with these cell culture observations, steady-state levels of CCL18 mRNA were readily found in SF PMN from patients with RA in the absence of detectable cell-associated CCL18. Its synthesis, however, was inducible on incubation of SF PMN with IL-10. Previous studies showed that TNF-α is able to prime PMN for subsequent downstream events [33]. Hence it is reasonable to conclude that CCL18 production by PMN requires two independent signals, one for mRNA expression, provided by TNF-α, and the other for protein synthesis, mediated by IL-10; these two cytokines are readily available in SF [31]. In DC cultures, differential regulation of CCL18 mRNA and protein expression has been attributed to changes in maturation [34,35]. It is unclear whether additional maturation also accounts for the differential regulation of CCL18 in PMN, because this cell type is considered to be terminally differentiated. However, Iking-Konert and colleagues [36] suggested that SF PMN can further differentiate into a dendritic-like phenotype. Another speculative explanation for the regulation of CCL18 synthesis in PMN might involve microRNAs [37].

Because TNF-α-dependent production of CCL18 by PMN was inducible by means of cell–cell contact with endothelial cells, IL-10 may not be the only inducer for CCL18 synthesis. It has previously been shown that recognition of β2-integrin/ICAM-1 on fibroblast-like synoviocytes induced MIP-1α expression in SF PMN [38]. Whether a similar ICAM-1-related mechanism accounts for the endothelial cell-mediated induction of CCL18 in PMN is unknown.

IL-10 alone was unable to induce CCL18 in blood PMN, and its release was not enhanced by stimulating PMN with IL-10 in combination with TNF-α. The apparent difference in IL-10 responsiveness between SF PMN and blood PMN from patients with RA may result from different expression patterns of the IL-10 receptors (IL-10R). It was shown that circulating PMN express high levels of the IL-10R2 and low levels of IL-10R1 [39]. Increasing IL-10R1 by stimulation with lipopolysaccharide coincided with an increased responsiveness of PMN to IL-10. The increased responsiveness in SF PMN may be induced by soluble factors present in SF, because preincubation of blood PMN with SF supported the induction of CCL18 by IL-10 alone. In this context, CCL18 production was synergistically enhanced when PMN were stimulated with IL-10 in combination with vitamin D_3_. These findings suggest that the high CCL18 levels in SF from RA patients [19] may be induced synergistically, as previously shown for monocytes cultured with IL-10 and SF from patients with RA [35]. The unidentified stimulating activity in SF [35] may be attributed to vitamin D_3_, because SF macrophages were shown to be able to produce vitamin D_3 _in cell culture experiments [40]. As IL-10 and vitamin D_3 _are known to have anti-inflammatory properties [41,42], the release of CCL18 induced by these mediators might contribute to a suppression of joint inflammation.

CCL18 has already been recognized in tissues and joints of patients with RA. In particular, high levels of CCL18 were measured in SF, and immunostaining of tissue sections revealed CCL18 production in perivascular regions of the synovia in CD68-positive monocytes/macrophages [19]. Furthermore, CCL18 mRNA and protein were found in DCs, generated *in vitro *from monocytes of RA patients [43], implying that high levels of CCL18 in SF originate from macrophages as well as DCs. The contribution of PMN to the accumulation of CCL18 in SF is difficult to judge because the cells have a low production rate for cellular mediators. However, because PMN are the majority of cells recruited into SF, the large number of PMN may compensate for their low production rate at the cellular level [44]. PMN may therefore be considered a cellular source of CCL18, contributing at least partly to the levels of CCL18 found in diseased joints.

Microarray analysis was performed to compare CCL18 mRNA expression in SF PMN from patients with RA with expression levels of other chemokines. The results show a characteristic profile of highly regulated chemokine mRNAs that form a group of four upregulated chemokines, namely CCL18, CCL3, CXCL8 and CXCL10, and one downregulated chemokine, namely CCL21. It is therefore unlikely that SF PMN favor the formation of ectopic germinal centers, in which increased levels of CCL21 were detected [45]. In contrast with the transcriptional program of terminal granulocytic differentiation [46], chemokines in SF PMN from patients with RA were not upregulated in parallel with their receptors. With the exception of CXCR4, CCRL2 and CCR5 mRNAs, most chemokine receptors were downregulated in SF PMN, supporting the notion of a differential modulation of chemokine receptors and their ligands during chronic inflammation. These findings suggest that SF PMN from patients with RA develop into a stage of unresponsiveness through the downregulation of chemokine receptors. Possible mechanisms accounting for the suppression of chemokine responsiveness may include ligand-induced receptor internalization and TNF-α-mediated proteolytic degradation of chemokine receptors [47]. However, it is also possible that some of the SF PMN with low levels of CXCR1, for example, develop into the recently described long-lived PMN with the capability of migrating across the endothelium in a retrograde direction [48]. In contrast, large numbers of CCR5 molecules on PMN have been attributed to a sequestration of chemokines, which may help to resolve the local inflammation [49]. Upregulation of CCR5 mRNA in SF PMN may therefore contribute to a regulation of the local chemokine response in diseased joints. The role of CCRL2 on SF PMN remains obscure, because no ligand has yet been identified [50].

Microarray analysis of blood PMN from patients with X-linked chronic granulomatous disease revealed increased mRNA levels for only two chemokines, namely CXCL8 and CXCL1 [51]. When compared with the chemokine expression profile in SF PMN from patients with RA, the data indicate a disease-specific expression of chemokines in PMN. This conclusion is supported, at least in part, by the results of Sukumaran and colleagues [52], who reported the upregulated expression of chemokine genes encoding CXCL1, CXCL2, CXCL3, CXCL8, CCL3, CCL4 and CCL20 after infection of PMN with *Anaplasma phagocytophilum*. However, no mRNA expression was observed for CCL18 and CXCL10 in PMN infected with *A. phagocytophilum*.

## Conclusion

This is the first study showing that SF PMN from patients with RA is a cellular source of CCL18. Its production by PMN seems to be tightly regulated at the levels of mRNA expression and protein synthesis. SF PMN from patients with RA exhibit a characteristic chemokine expression pattern resembling the upregulation of CCL3, CCL18, CXCL8 and CXCL10 mRNAs and the downregulation of CCL21 mRNA. Blockade of CCL18 expression by anti-TNF-α antibodies identifies CCL18 as an additional target for anti-TNF-α therapy in patients with RA.

## Abbreviations

APC = allophycocyanin; DC = dendritic cell; DMEM = Dulbecco's modified Eagle's medium; ELISA = enzyme-linked immunosorbent assay; FCS = fetal calf serum; IL = interleukin; PBMC = peripheral blood mononuclear cells; PBS = phosphate-buffered saline; PMN = polymorphonuclear neutrophils; RA = rheumatoid arthritis; RT-PCR = reverse transcriptase polymerase chain reaction; SF = synovial fluid; TNF = tumor necrosis factor.

## Competing interests

The authors declare that they have no competing interests.

## Authors' contributions

JA performed research and supported the paperwork including figures and text. MB performed the microarray analysis. HS-K and TN, as physicians at the local hospital, prepared the samples from patients with RA. SR supervised the microarray analysis. MR and JRK provided advice on experimental design. HUB supervised the experimental work and wrote the paper. All authors read and approved the final manuscript.
